# Race and sex differences in ROS production and SOD activity in HUVECs

**DOI:** 10.1371/journal.pone.0292112

**Published:** 2023-10-04

**Authors:** Sara E. Mascone, Katherine I. Kim, William S. Evans, Steven J. Prior, Marc D. Cook, Sushant M. Ranadive

**Affiliations:** 1 Department of Kinesiology, School of Public Health, University of Maryland, College Park, MD, United States of America; 2 Department of Kinesiology, Hairston College of Health and Human Sciences, North Carolina Agricultural and Technical State University, Greensboro, NC, United States of America; National Institutes of Health, UNITED STATES

## Abstract

Black individuals and men are predisposed to an earlier onset and higher prevalence of hypertension, compared with White individuals and women, respectively. Therefore, the influence of race and sex on reactive oxygen species (ROS) production and superoxide dismutase (SOD) activity following induced inflammation was evaluated in female and male human umbilical vein endothelial cells (HUVECs) from Black and White individuals. It was hypothesized that HUVECs from Black individuals and male HUVECs would exhibit greater ROS production and impaired SOD activity. Inflammation was induced in HUVEC cell lines (n = 4/group) using tumor necrosis factor-alpha (TNF-α, 50ng/ml). There were no between group differences in ROS production or SOD activity in HUVECs from Black and White individuals, and HUVECs from Black individuals exhibited similar SOD activity at 24hr compared with 4hr of TNF-α treatment (p>0.05). However, HUVECs from White individuals exhibited significantly greater SOD Activity (p<0.05) at 24hr as compared to 4hr in the control condition but not with TNF-α treatment (p>0.05). Female HUVECs exhibited significantly lower ROS production than male HUVECs in the control condition and following TNF-α induced inflammation (p<0.05). Only female HUVECs exhibited significant increases in SOD activity with increased exposure time to TNF-α induced inflammation (p<0.05). HUVECs from White individuals alone exhibit blunted SOD activity when comparing control and TNF-α conditions. Further, compared to female HUVECs, male HUVECs exhibit a pro-inflammatory state.

## Introduction

Cardiovascular disease (CVD) is the leading cause of death in the United States, and race and sex are well established mediators of CVD risk [1]. CVD is thought to be a culmination of chronic inflammation, oxidative stress, and vascular dysfunction, termed the vascular health triad [2–5]. Vascular function describes the vasodilatory capacity of a blood vessel. Vasodilation is largely attributed to endothelial cells, which compose the innermost layer of blood vessels and release vasodilatory substances, such as nitric oxide (NO) [6–8]. Endothelial cells are also impacted by inflammation and mediate oxidative stress via NO release and subsequent NO-mediated reactive oxygen species (ROS) scavenging [3, 6, 7, 9, 10]. Because endothelial cells are implicated in vascular dysfunction, inflammation, and oxidative stress, they are often used as a model to investigate cardiovascular disease mechanisms. Human umbilical vein endothelial cells (HUVECs) are harvested from the umbilical cord following birth, and the sex of the baby and the mother’s age and health status recorded. While not directly possible in many *in vivo* models, investigation of mechanisms of cardiovascular disease via treatment with inflammatory cytokines [11], vaccines [12], or differing shear stresses [13] is possible in HUVECs.

Importantly, compared with HUVECs from White individuals, HUVECs from Black individuals have exhibited greater inflammation, as indicated by higher NO levels and higher Interleukin-6 (IL-6) concentrations [14]. HUVECs from Black individuals have also exhibited greater expression of ROS producing proteins, suggesting higher ROS production [14–16]. Interestingly, HUVECs from Black individuals have exhibited higher total antioxidant capacity (TAC) but lower superoxide dismutase (SOD) activity, suggesting a greater overall ability to clear or remove excess ROS [11, 13, 14]. Taken together, basally or at rest, HUVECs from Black individuals exhibit greater inflammation, greater ROS production, and higher ROS clearance capacity compared with HUVECs from White individuals. However, to the best of our knowledge, there is limited data regarding the role of race in oxidative stress (ROS production and clearance) following induced inflammation in HUVECs.

Sex differences in inflammatory response following induced inflammation have also been noted in HUVECs [17]. In umbilical cord blood to male and female babies, there is no difference in estrogen exposure, however, male babies are exposed to greater testosterone concentrations than female babies [18]. Interestingly, when primed with testosterone, but not estradiol, for 48 hours, HUVECs from both male and female individuals exhibit greater inflammatory responses to tumor necrosis factor alpha (TNF-α) [17]. However, when primed with testosterone derivates for 1 hour before TNF-α stimulation, HUVECs exhibit a reduced inflammatory response to TNF-α [19]. Thus, exposure time to sex hormones may play an important role in inflammatory responses. However, there is limited data regarding the role of sex in oxidative stress (ROS production and clearance) following induced inflammation.

Therefore, the purpose of the current study was to investigate the influence of race and sex on ROS production and SOD activity in HUVECs following induced inflammation. We hypothesized that HUVECs from Black individuals would exhibit greater ROS production and lower SOD activity than HUVECs from White individuals following induced inflammation. Further, we hypothesized that female HUVECs would exhibit lower ROS production and greater SOD activity than male HUVECs following induced inflammation. TNF-α was utilized to induce inflammation due to its role in inflammation and cytokine production in the human body [20] and its efficacy in previous studies [11, 17].

## Methods

### Ethical approval

All procedures were approved by the Institutional Review Board at the University of Maryland- College Park (IRB# 1713185) and conformed to standards set by the Declaration of Helsinki. All protocols were performed on anonymized, commercially obtained HUVEC samples, making the protocol exempt from direct human subjects informed consent, as determined by the IRB.

### Human umbilical vein endothelial cell lines

**Fig 1:**
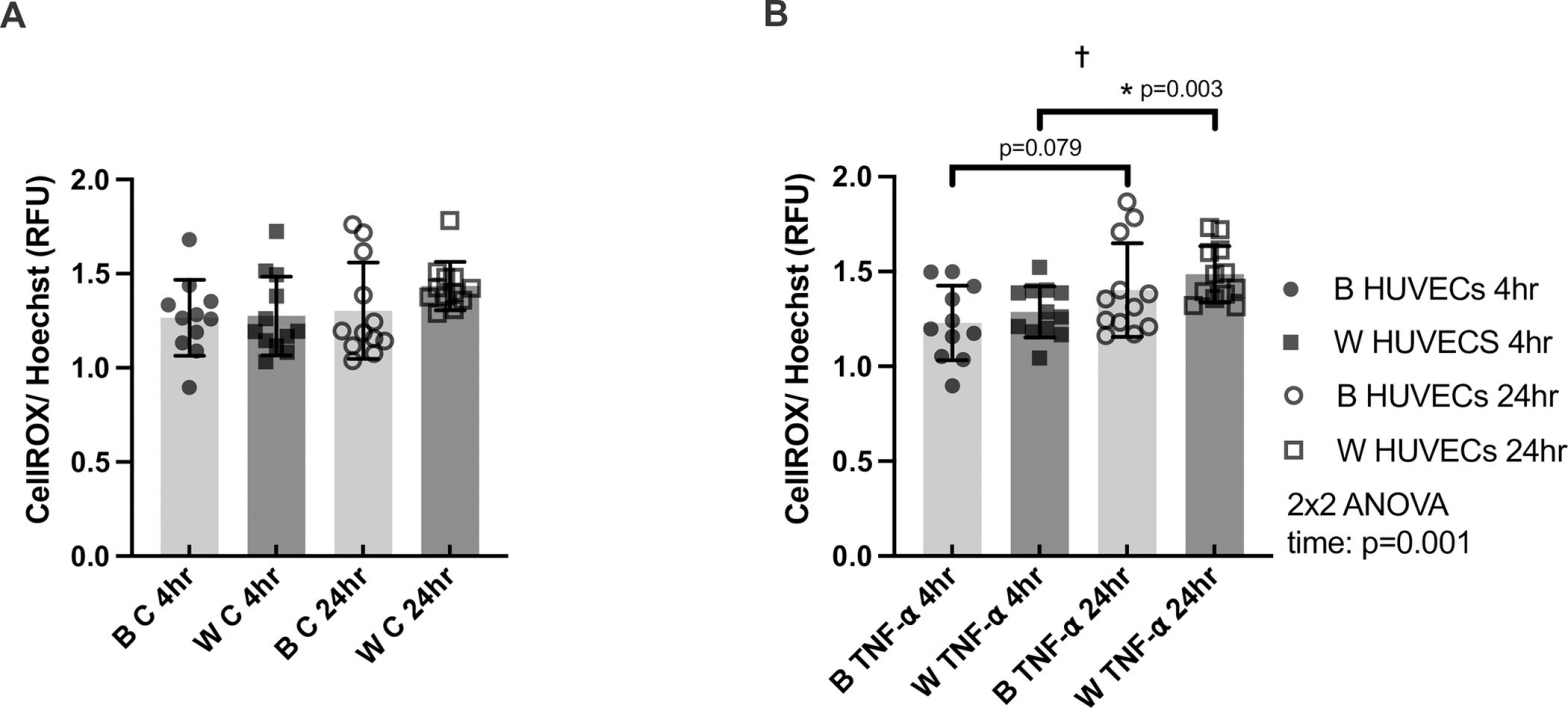
CellROX/ Hoechst (ROS production normalized to living cells) in HUVECs from Black (B) and White (W) individuals at 4 hours and 24 hours of time-matched control (panel A) and TNF-α treatment (panel B). n = 11 for Control 4hr B HUVECs and TNF-α 4hr B HUVECs, n = 12 for all other groups and times. * denotes p<0.05 from post-hoc t-tests and significant model effects are denoted by † (time), # (sex), or ^ (interaction).

**Fig 2:**
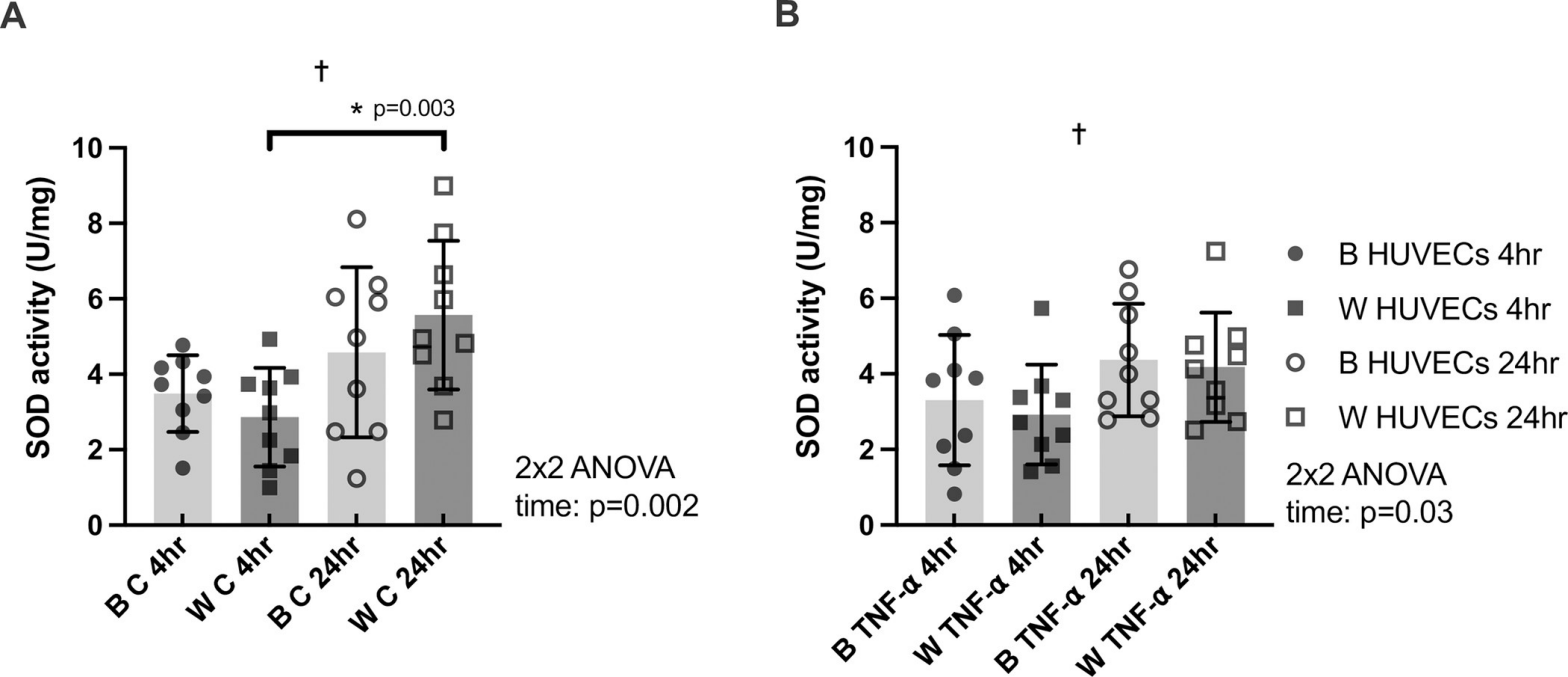
SOD activity normalized to protein content (U/mg) in HUVECs from Black (B) and White (W) individuals at 4hr and 24hr for control (panel A) and TNF-α treatment (panel B). n = 9 for all groups and times. * denotes p<0.05 from post-hoc t-tests and significant model effects are denoted by † (time), # (sex), or ^ (interaction).

**Fig 3:**
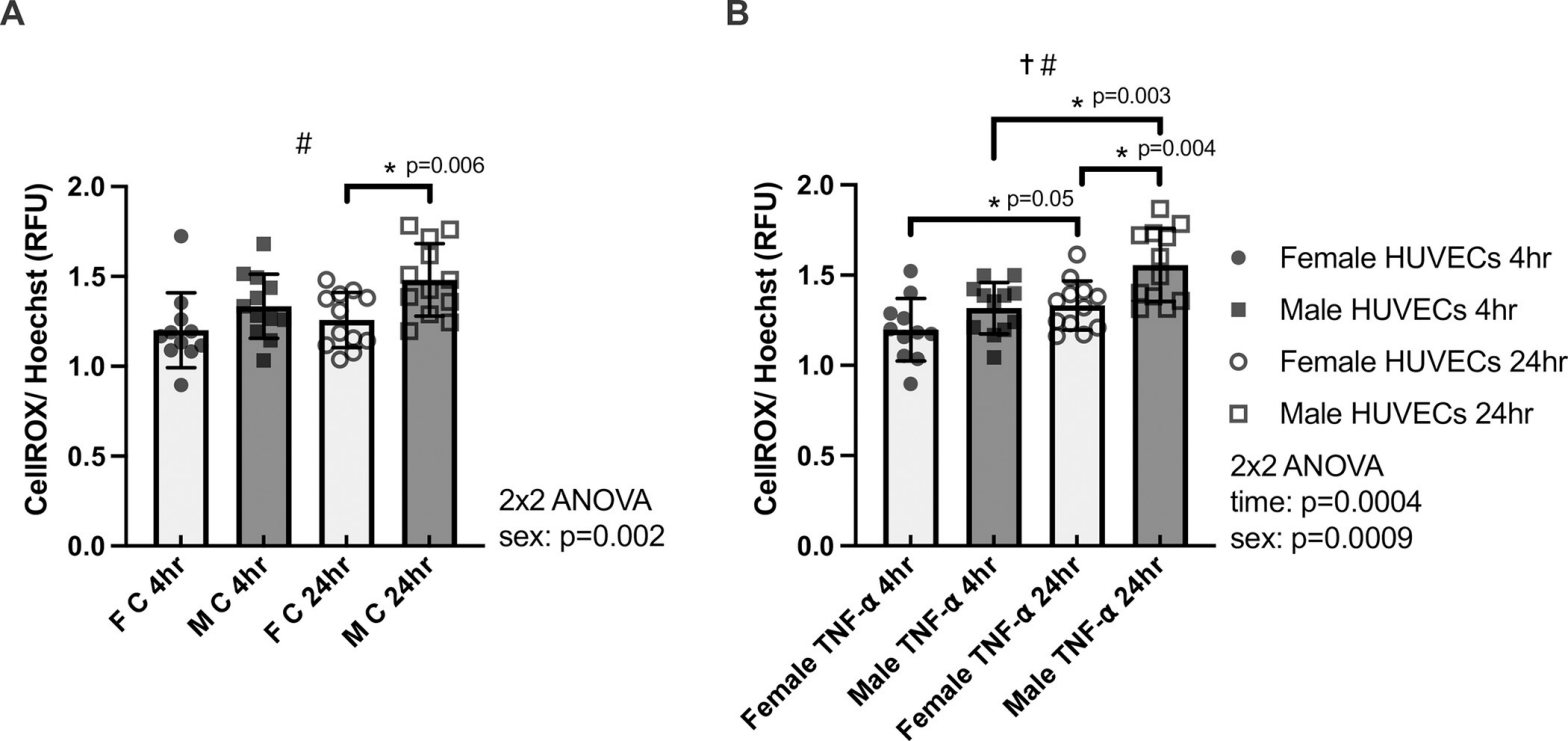
CellROX/ Hoechst (ROS production normalized to living cells) in female (F) and male (M) HUVECs at 4 hours and 24 hours of time-matched control (panel A) and TNF-α treatment (panel B). n = 11 for Control 4hr F HUVECs and TNF-α 4hr F HUVECs, n = 12 for all other groups and times. * denotes p<0.05 from post-hoc t-tests and significant model effects are denoted by † (time), # (sex), or ^ (interaction).

**Fig 4:**
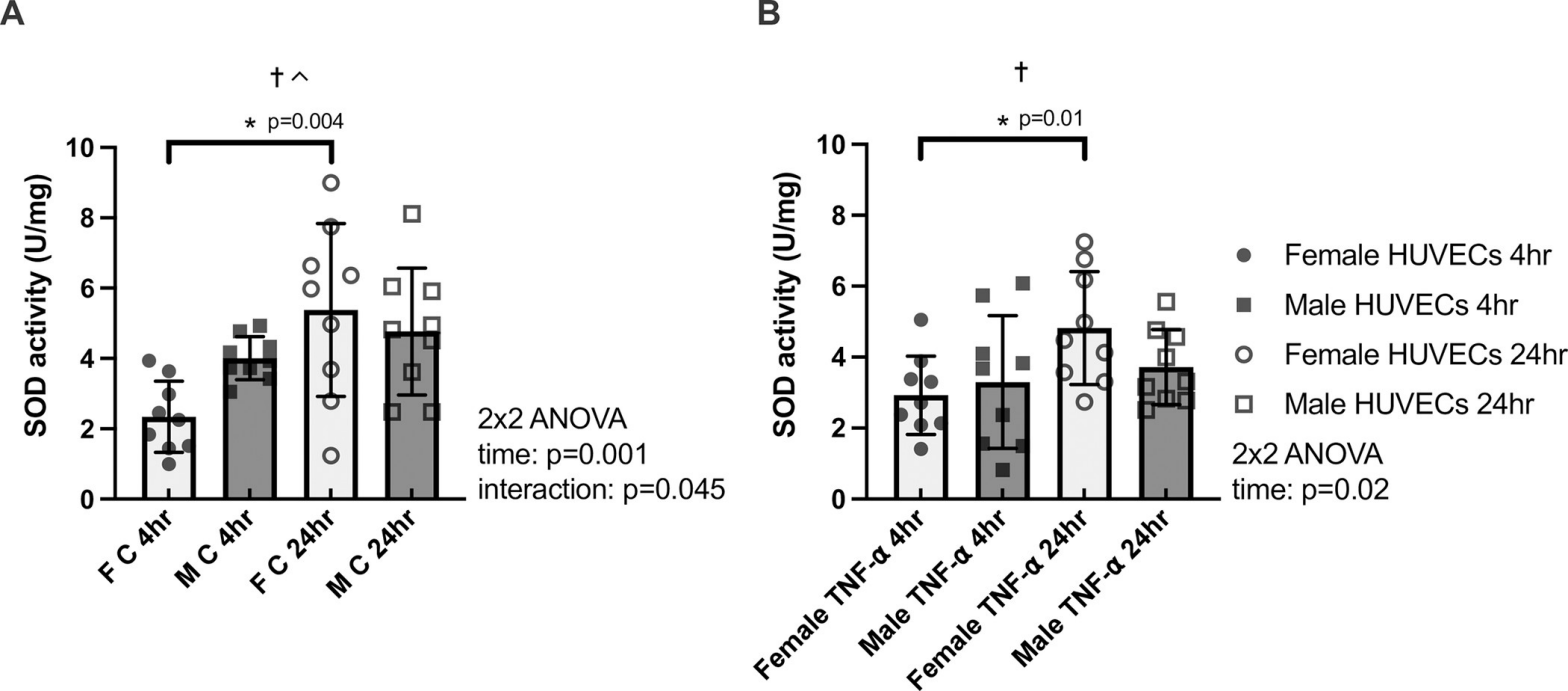
SOD activity normalized to protein content (U/mg) in female (F) and male (M) HUVECs at 4 hours and 24 hours of time-matched control (panel A) and TNF-α treatment (panel B). n = 9 for all groups and times. * denotes p<0.05 from post-hoc t-tests and significant model effects are denoted by † (time), # (sex), or ^ (interaction).

HUVECs were obtained from a commercial company, Lonza, from young, healthy donors free of overt disease or pregnancy complications. Eight total cell lines were obtained: two cell lines of each race and each sex (n = 4 for each racial group and sex group). For analyses of HUVECs from Black and White individuals (Figs 1 and 2), each racial group includes two female and two male cell lines (n = 9–12 per time point and condition). For analyses of female and male HUVECs (Figs 3 and 4), each sex group includes two cell lines from each racial group (Black and White; n = 9–12 per time point and condition). The inclusion of different sexes in the by race analyses and different races in the by sex analyses was done to balance the impact of different races or sexes. For SOD activity, one black, female cell line and one white, male cell line were not analyzed due to poor cell growth, leading to n = 9 for all SOD activity data. For female CellROX/ Hoechst data, one of the triplicate data points for one of the black, female cell lines had poor Hoechst absorbances, leading to n = 11.

### Inflammatory stimulus

TNF-α, a pro-inflammatory cytokine, was used to induce inflammation in the HUVECs. First, optimization experiments were performed to determine the TNF-α concentration needed to elicit inflammation without unnecessary cell death. Pooled HUVECs (separate from those used in experiments) in endothelial basal media and 2% fetal bovine serum were exposed to increasing concentrations of TNF-α for 24 hours and cells were visually analyzed for cell death and detachment. ROS production was measured via CellROX green assay and cell count was assessed via Hoechst assay (Thermo Scientific- ThermoFisher Scientific; Waltham, Massachusetts). Based on the results, the experimental TNF-α concentration was determined, as the greatest ROS production per viable cells occurred at 50 ng/ml.

### HUVECs experimental overview

All experiments were performed in triplicate on Black, White, female, and male HUVECs treated identically and cultured in parallel. Based on estimated between group means and standard deviations of n = 6/group from TNF-α stimulation in Brown et al. [11], a power calculation for the present study determined the effect size for SOD activity was 1.09 with α = 0.05, 1-β = 0.80. HUVECs were received at passage 1 (P1) and stored in liquid nitrogen. HUVECs were thawed, grown to ~80% confluence, and stored in P2 aliquots. All experiments were performed in P3-5 HUVECs. HUVECs were grown to 80% confluence before being cultured in either (1) endothelial growth medium (Lonza; Basel, Switzerland) and 2% fetal bovine serum (time-matched control) or (2) endothelial growth medium, 2% fetal bovine serum, and the experimental TNF-α concentration (50ng/ml) in 96-well (10,000 cells/well seeded at least 18 hours prior to time 0 of experimentation) or six-well plates (60,000 cells/well seeded at least 18 hours prior to time 0 of experimentation). Six-well plate cell lysate samples were collected at 4 hours and 24 hours following time-matched control or TNF-α stimulation. For cell lysate collection, six-well plates were placed on ice and 20mM HEPES buffer containing 1mM EGTA, 210mM mannitol, and 70mM sucrose was pipetted onto the cells. After scraping with a sterile bent pipette tip, cell lysate samples were collected in Eppendorf tubes and rotated for 20 minutes at 4°C. The samples were then centrifuged at 1500xg for 20 minutes at 4°C. The supernatant was removed and stored at -80°C for future assay. To determine protein concentration, Pierce BCA protein assays (Thermo Scientific- ThermoFisher Scientific; Waltham, Massachusetts) were performed on cell lysate samples according to manufacturer’s instructions. Briefly, a series of known bovine serum albumin (BSA) standards were prepared to achieve BSA concentrations from 2000ug/ml to 25 ug/ml. Next, the BCA working reagent was prepared by mixing 50 parts of BCA Reagent A to 1 parts of BCA reagent B. Then, 25ul of each BSA standard and unknown were pipetted in duplicate into a 96 well plate followed by 200 ul of the BCA working reagent. Following adequate shaking of the microplate, the microplate was incubated at 37°C for 30 minutes prior to absorbance readings at 562nm. BCA protein concentrations were then determined from the standard curve from the BSA standards, and protein concentration values were averaged for unknown samples.

### ROS production

To determine ROS production at 4 hours and 24 hours following time-matched control or TNF-α stimulation, CellROX green assays (Invitrogen- ThermoFisher Scientific; Waltham, Massachusetts) were performed according to manufacturer’s instructions. Cells were passed into 96 well plates with EGM-2 and 2% fetal bovine serum media and allowed to grow to 80% confluence prior to experimentation. On the day of experimentation, media with or without 50 ng/ml TNF-α was placed on the cells and allowed to incubate for 4 or 24 hours (separate plates used for each time condition). For the fluorescence assay, 5 μM of CellROX Reagent and 1 μM Hoechst in EGM-2 and 2% fetal bovine serum was placed on the cells (master mix contained 4.8ml of EGM-2, 4.8μl of Hoescht, and 7.2μl of CellROX). Following a 30 minute incubation, the medium was removed and cells were washed three times with PBS. Fluorescence was immediately performed via a micro-plate reader (Synergy H1 Hybrid Reader; BioTek, Winooski, VT) with excitation at 485nm and emission at 520nm for CellROX and excitation at 361nm and emission at 486nm for Hoechst.

Cell count was assessed via Hoechst assay (Thermo Scientific- ThermoFisher Scientific; Waltham, Massachusetts) performed according to manufacturer’s instructions. To determine the ROS produced per viable cells, a CellROX/ Hoechst ratio was calculated and used to compare ROS production between race and sex groups.

### SOD activity

SOD activity was subsequently determined in cell lysate samples via a commercially available SOD activity assay kit according to manufacturer’s instructions (Cayman Chemical; Ann Arbor, Michigan). Through xanthine oxidase dependent superoxide production, this assay indexes activity of all three SODs: SOD1 (cytoplasmic), SOD2 (mitochondrial), and SOD3 (extracellular). One unit of SOD is the amount of SOD needed to elicit 50% dismutation of superoxide [21].

On the day of assay, 200μL of the diluted radical detector was added to each well in the 96 well plate. Then, 10μL of serially diluted standard or diluted sample was added to each well. 20μL of xanthine oxidase was added to each well as quickly as possible. Following a 30 minute incubation on a plate shaker, absorbance was read between 440 and 460 nm using a plate reader. The absorbance of the known standards was used to create the absorbance-concentration equation. The determined equation was then used to calculate the concentration of each sample based on absorbance. Standards and samples were analyzed in duplicate and averaged across duplicates. SOD activity is normalized to protein content (U/mg).

### Statistical analysis

The present study evaluated the impact of race and sex on inflammatory responses and ROS clearance capacity in HUVECs following induced inflammation. All data were assessed for normality and outliers via Shapiro-Wilk test for normality and the ROUT 1% method (1% = the false discovery rate of outliers) [22], respectively. Two-way ANOVAs with factors of race and time (4 hours and 24 hours) or sex and time (4 hours and 24 hours) were performed separately for the time-matched control and TNF-α conditions. Post-hoc t-tests were performed on any significant model effects. Data are presented as mean (SD) and effect sizes (Hedges’s g_s;_ Formula: Hedges’s g_s_ = $\frac{{mean}_{group\;1}+{mean}_{group\;2}}{{standard\;deviation}_{pooled}}$) of significant findings were calculated using a supplemental effect size spreadsheet [23]. Effect sizes represent the magnitude of difference between group mean in terms of standard deviations, whereby an effect size of g_s_ = 1 represents a 1 standard deviation difference in means between two compared groups. For Hedges’s g_s_, an effect size of 0.2 is considered a small effect size, 0.5 is considered a medium effect size, and 0.8 is considered a large effect size. All statistical analyses were performed in GraphPad Prism v9 (San Diego, CA).

## Results

### No race differences in ROS production in HUVECs

For ROS production in the control condition, there were no significant model effects, suggesting similar ROS production within and between races during the time-matched control (**Fig 1A**). For ROS production in the TNF-α condition, there was a significant main effect of time alone (p = 0.0014; **Fig 1B**). In HUVECs from Black individuals, the effect size of ROS production at 24hr as compared with 4hr of TNF-α stimulation was Hedges’s g_s_ = 0.73 (p = 0.079). In HUVECs from White individuals, the effect size of ROS production at 24hr as compared with 4hr of TNF-α stimulation was Hedges’s g_s_ = 1.37 (p = 0.0025).

### Race differences in SOD activity in HUVECs

For SOD activity normalized to protein content (U/mg), there was a significant effect of time for both the control (**Fig 2A**) and TNF-α conditions (**Fig 2B**) (p = 0.0022 and p = 0.027, respectively). However, further analyses revealed that SOD activity normalized to protein content in HUVECs from Black individuals was similar between time points for both conditions (SOD activity (U/mg)- B control 4hr vs 24hr: p>0.05; TNF-α 4hr vs 24hr: p>0.05). SOD activity normalized to protein content in HUVECs from White individuals was significantly greater at 24hr as compared to 4hr in the control condition alone (SOD activity (U/mg)- W control 4hr vs 24hr: p = 0.0034, Hedges’s g_s_ = 1.54; TNF-α 4hr vs 24hr: p>0.05).

### Sex differences in ROS production in HUVECs

For ROS production in the control condition, there was a significant main effect of sex (p = 0.0020, time effect p = 0.069, **Fig 3A**). Further investigation revealed female HUVECs exhibited significantly lower ROS production than male HUVECs at 24hr in the control condition (p = 0.0060, Hedges’s g_s_ = 1.20). For ROS production in the TNF-α condition, there were significant effects of time and sex (p = 0.0004 and p = 0.0009, respectively; **Fig 3B**). Both female and male HUVECs exhibited greater ROS production at 24hr of TNF-α stimulation when compared to 4hr of TNF-α stimulation (female: p = 0.050, Hedges’s g_s_ = 0.81; male: p = 0.0030, Hedges’s g_s_ = 1.34). Interestingly, female HUVECs exhibited significantly lower ROS production than male HUVECs at 24hr for the TNF-α condition (p = 0.0040, Hedges’s g_s_ = 1.29).

### Sex differences in SOD activity in HUVECs

For SOD activity normalized to protein content in the control condition, there were significant effects of time and a significant interaction (p = 0.0014 and p = 0.045, respectively; **Fig 4A**). For SOD activity normalized to protein content in the TNF-α condition, there was a significant effect of time (p = 0.022; **Fig 4B**). Further within sex analysis revealed female HUVECs exhibited significantly greater SOD activity normalized to protein content at 24hr as compared with 4hr for both conditions (control: p = 0.0035, Hedges’s g_s_ = 1.52; TNF-α: p = 0.0098, Hedges’s g_s_ = 1.31), and male HUVECS exhibited similar SOD activity (U/mg) between time points for both conditions (p>0.05).

## Discussion

The novel findings of the study are: 1) HUVECs from White individuals alone experienced an increase in SOD activity with increased growth time that was abolished with TNF-α treatment; 2) female HUVECs exhibited significantly lower ROS production than male HUVECs in the control and TNF-α conditions; and 3) female HUVECs exhibited significantly greater SOD activity with increased exposure time to TNF-α.

### No racial differences in ROS production

Previous studies have shown that HUVECs from Black individuals have significantly higher basal protein expression of various superoxide-producing NADPH oxidases, suggesting higher basal ROS production [13–15]. Interestingly, as compared with HUVECs from White individuals, HUVECs from Black individuals also exhibit greater endothelial nitric oxide synthase (eNOS) expression. Increased eNOS expression yet a concomitant greater ROS production, as in seen in previous literature, may indicate decreased NO bioavailability, increased NO-related clearance of superoxide, or increased ROS production in HUVECs from Black individuals [13–15]. However, the findings from the current study are not in agreement with previous literature, potentially due to the magnitude of the inflammatory stimulus used, priming of HUVECs from Black individuals from chronic stressors to the mother, or differential inflammatory path activity or activation between HUVECs from White and Black individuals. In the present study, HUVECs from both the races exhibited increases in ROS production at 4hr and 24hr of TNF-α treatment with no differences between races basally. Plausibly the magnitude of ROS production, based on the effect sizes, was greater in HUVECs from White individuals (Hedges’s g_s_ = 1.37) as compared to HUVECs from Black individuals (Hedges’s g_s_ = 0.73).

A plausible explanation for discrepancies in the present study as compared to the previous literature is the differential inflammatory pathways in HUVECs from Black and White individuals. Specifically, recent studies suggest HUVECs from Black individuals have greater inflammatory responses (greater metalloproteinase-2 and endothelial microparticle release) to TNF-α stimulation as compared to HUVECs from White individuals [11, 24]. HUVECs from Black individuals have also exhibited greater C-reactive protein (CRP) receptor expression than HUVECs from White individuals before and after induced inflammation, potentially suggesting a greater ability to respond to CRP binding and inflammation generally [25]. Taken together, the current and previous findings suggest differential inflammatory pathways in HUVECs from Black and White individuals, with HUVECs from Black individuals potentially exhibiting greater inflammation and potentially causing greater counteractive changes in oxidative stress–e.g., a greater responsiveness in terms of greater NO concentrations, greater eNOS expression, greater CRP receptor expression, and greater responses to induced inflammation and laminar shear stress [13–15, 24, 25].

As race is a social construct, a second potential explanation for the discrepancies in response to stressors in the present study versus previous studies could be social factors (beyond the scope of this study) whereby socially, Black individuals experience more chronic stress (racism, socio-economic, and housing-related stress, for example) than White individuals [26], and thus are more “primed” and less responsive to added stressors [27]. While these previous findings are in humans, perhaps a similar phenomenon occurs physiologically, whereby, when HUVECs are treated with similar concentrations of TNF-α, the impact of those concentrations may be different at the cellular level in Black and White individuals.

### Racial differences in SOD activity

SOD is an enzyme that primarily attenuates ROS levels by clearing superoxide. In the present study, when comparing the 4hr and 24hr control conditions, HUVECs from White individuals, but not HUVECs from Black individuals, exhibited greater SOD activity normalized to protein content. This may be due to the previously mentioned differential impact of the magnitude of the inflammatory stimulus used (i.e. HUVECs from Black individuals may be primed to respond to the inflammatory stimulus used while HUVECs from White individuals may not be as primed). The increase in SOD activity in HUVECs from White individuals in the control condition was absent in the TNF-α condition, suggesting a reduction or attenuation of the normally increased SOD activity when exposed to TNF-α in HUVECs from White individuals alone. In the present study, SOD activity in HUVECs from Black individuals did not significantly change with time in the control or TNF-α condition. Indeed, HUVECs from Black individuals have exhibited significantly lower SOD activity normalized to protein content basally and following 4 hours of TNF-α exposure when compared with HUVECs from White individuals [11, 13, 14]. Yet, HUVECs from Black individuals have also exhibited significantly greater total antioxidant capacity than HUVECs from White individuals basally [14]. Taken together, it is plausible HUVECs from Black individuals may be primed to respond to inflammatory stimuli due to a higher capacity to clear ROS from other non-SOD1 and SOD2 antioxidant sources (glutathione peroxidase, uric acid, SOD3 [extracellular]). The similar ROS production and SOD activity seen in HUVECs from Black individuals in the current study is plausibly explained by greater total antioxidant capacity in HUVECs from Black individuals. However, young, healthy Black individuals exhibit greater plasma and circulating immune cell oxidative stress *and* total antioxidant capacity and SOD activity [14, 28, 29].

### Sex differences in ROS production and SOD activity

Sex differences in ROS production and SOD activity may be due to 1) male HUVECs being primed to exhibit a pro-inflammatory state due to higher androgen exposure in the umbilical cord [18], 2) male HUVECs having a dampened antioxidant response to TNF-α stimulation that may be sex chromosome related [30], and/or 3) male HUVECs having augmented cellular stress responses [31]. In the present study, male HUVECs exhibited significantly greater ROS production than female HUVECs basally and following induced inflammation. With SOD activity normalized to protein content, only female HUVECs exhibited significantly greater SOD activity (U/mg) at 24hr when compared with 4hr for both conditions, further supporting better ROS clearance capabilities via increasing SOD activity in female HUVECs.

Sex differences in ROS production and SOD activity may be due to differences in androgen actions, total antioxidant capacity, or cellular stress responses. The concentration of estrogen in umbilical cord blood does not differ between fetal sex, and, thus, male and female HUVECs likely experience similar estrogen concentrations [18]. Lower actions of androgens in female HUVECs as compared to male HUVECs could explain the lower ROS production and higher SOD activity in female HUVECs. In a previous study, androgen exposure primed the inflammatory impacts of TNF-α exposure in female and male HUVECs, and umbilical cord blood has shown sex differences in testosterone levels, with male umbilical cords exhibiting higher testosterone concentrations [17, 18]. Specifically, androgens have been shown to increase vascular adhesion molecule expression and monocyte adhesion, molecules and events implicated in CVD progression [17]. It is plausible the greater androgen concentrations in umbilical cord blood of male HUVECs ‘primes’ a more pro-inflammatory state in male HUVECs as compared with female HUVECs. Whereby, exposure to a subsequent inflammatory stimulus, such as TNF-α, results in greater oxidative stress (heightened ROS production) and impaired SOD activity in male HUVECs as compared with female HUVECs. However, there are noted sex-specific effects of circulating androgens in men and women [32]; some evidence suggests testosterone is vasodilatory and antioxidative in men [33] while other evidence suggests no cardiovascular protection from testosterone in men [34]. The effects of testosterone in men and women are still being studied. Thus, the present findings and the role of sex in the actions of testosterone warrant further investigation.

The present study’s results suggest a greater ability to clear ROS via greater SOD activity following induced inflammation in female HUVECs, as indicated by lower ROS levels as compared to male HUVECs yet increased SOD activity in female HUVECs alone. Indeed, previous literature suggests about a quarter of endothelial cell transcripts for genes are influenced by sex [30]. Thus, the present findings may be due to sex-based differences in the expression of SOD or other antioxidants, but further research is warranted to explore this potential relationship. Further, following cellular stress in a previous study, male HUVECs exhibited greater ROS production, lower cell viability, lower angiogenesis, and lower NF-kB pathway activation than female HUVECs, suggesting a greater oxidative response and lower inflammatory response in male HUVECs [17, 31]. Taken together, the current results and previous literature suggest male HUVECs may exhibit a more pro-inflammatory state (greater ROS production, lower SOD activity, greater apoptosis, impaired angiogenesis) than female HUVECs.

### Considerations

These disparate physiological responses, especially in the HUVECs, could “*stem from disparities in socio-economic status*, *educational status*, *as well as other social determinants of health which a growing body of research has shown to be linked to structural racism*” faced by the mothers (beyond the scope of the current study) (p. H2372) [12]. The purpose of this study is not to suggest one race has inherently lower physiological function. On the contrary, it is recognized that ’race’ is a social construct- one with real, material consequences. The purpose of the current study is to present potential explanations of the observed results, which may be due to social determinants of health [12]. Therefore, one major limitation is the data does not take into consideration how various social determinants of health, including systemic racism faced by the mothers, could prime HUVEC responses in the present study [12]. Further, the maternal health environment (physical activity, social determinants of health factors, smoking status, social stressors, etc.) impacts the vascular health of the mother and, thus, the environment of HUVECs. Thus, it is important to consider the maternal health environment as a potential source of variation and differential responses in HUVECs [35].

### Limitations

The current study includes some limitations. First, only SOD activity was measured, and, thus, a complete picture of ROS clearance capacity was not fully captured. Second, HUVECs are subject to venous circulation as opposed to arterial circulation, meaning HUEVCs undergo different hemodynamics and are in a different environment than arterial endothelial cells. Importantly, HUVECs are a commonly used endothelial cell model [11, 12, 15, 25, 31], arterial endothelial cells can be hard to harvest from healthy individuals, and adequately harvested arterial endothelial cells often are from diseased or older individuals. Third, the current study did not measure levels of specific ROS or reactive nitrogen species, limiting the assumptions regarding alterations in oxidative stress. Lastly, because of the observed sex differences and within race divergences, sex and race likely confounded one another and require further research.

### Conclusions

HUVECs from Black and White individuals exhibit divergent responses to TNF-α-induced inflammation, with HUVECs from White individuals, but not HUVECs from Black individuals, exhibiting a blunted increase in SOD activity with increased exposure time to TNF-α. Interestingly, female and male HUVECS exhibited sex differences in ROS production and SOD activity; female HUVECs exhibiting significantly lower ROS production and significantly higher SOD activity than male HUVECs following TNF-α exposure, suggesting sex differences in susceptibility to induced inflammation in HUVECs. The current findings underly the importance of noting the race and sex (or indicating that race and sex are pooled if using pooled HUVECs) of HUVECs used in *in vitro* research.

## Supporting information

S1 Data (XLSX)
